# Ubiquitin Ligase HUWE1 Regulates Axon Branching through the Wnt/β-Catenin Pathway in a *Drosophila* Model for Intellectual Disability

**DOI:** 10.1371/journal.pone.0081791

**Published:** 2013-11-26

**Authors:** Joke Vandewalle, Marion Langen, Marlen Zschaetzsch, Bonnie Nijhof, Jamie M. Kramer, Hilde Brems, Marijke Bauters, Elsa Lauwers, Mohammed Srahna, Peter Marynen, Patrik Verstreken, Annette Schenck, Bassem A. Hassan, Guy Froyen

**Affiliations:** 1 Human Genome Laboratory, VIB Center for the Biology of Disease, Leuven, Belgium; 2 Human Genome Laboratory, Department of Human Genetics, KU Leuven, Leuven, Belgium; 3 Laboratory of Neurogenetics, VIB Center for the Biology of Disease, KU Leuven, Leuven, Belgium; 4 Department of Human Genetics, Nijmegen Centre for Molecular Life Sciences, Donders Institute for Brain, Cognition and Behaviour & Radboud University Medical Center, Nijmegen, The Netherlands; 5 Laboratory for Neurofibromatosis Research, Department of Human Genetics, KU Leuven, Leuven, Belgium; 6 Laboratory of Neuronal Communication, VIB Center for the Biology of Disease, KU Leuven, Leuven, Belgium; Dulbecco Telethon Institute at San Raffaele Scientific Institute, Italy

## Abstract

We recently reported that duplication of the E3 ubiquitin ligase *HUWE1* results in intellectual disability (ID) in male patients. However, the underlying molecular mechanism remains unknown. We used *Drosophila melanogaster* as a model to investigate the effect of increased HUWE1 levels on the developing nervous system. Similar to the observed levels in patients we overexpressed the *HUWE1* mRNA about 2-fold in the fly. The development of the mushroom body and neuromuscular junctions were not altered, and basal neurotransmission was unaffected. These data are in agreement with normal learning and memory in the courtship conditioning paradigm. However, a disturbed branching phenotype at the axon terminals of the dorsal cluster neurons (DCN) was detected. Interestingly, overexpression of *HUWE1* was found to decrease the protein levels of dishevelled (dsh) by 50%. As *dsh* as well as *Fz2* mutant flies showed the same disturbed DCN branching phenotype, and the constitutive active homolog of β-catenin, armadillo, could partially rescue this phenotype, our data strongly suggest that increased dosage of HUWE1 compromises the Wnt/β-catenin pathway possibly by enhancing the degradation of dsh.

## Introduction

Intellectual disability (ID) refers to cognitive impairment and affects ~2% of the population in developed countries. ID patients lack the necessary mental capabilities and adaptive skills required to live independently and rely on family members and other caretakers for help in daily life. ID therefore constitutes an important medical and socio-economical problem. Although numerous ID genes have been discovered during the last two decades, for many of them the molecular mechanism via which they contribute to the ID phenotype remains unknown. Particularly unexplored are mechanisms that underlie copy number gains in ID, increasing gene dosage, as most of the ID genes that have been investigated were studied in knockout animal models. 

We recently identified non-recurrent but overlapping microduplications at Xp11.22 in 12 unrelated families with mild to moderate non-syndromic ID [1,2]. The only coding gene present in the smallest region of overlap of these microduplications is *HUWE1*. The region also contains two miRNAs, but the identification of a partial *HUWE1* duplication harboring both miRNAs in healthy individuals strongly suggest that these do not contribute to ID phenotype [1]. These findings thus indicate that a modest increase in expression of *HUWE1*, 1.6- to 2.0-fold as observed in patients, is sufficient to cause non-syndromic cognitive impairment. Since all patients of the reported 12 families show only mild to moderate ID irrespective of the size of the duplication, *HUWE1* appears to be the main gene underlying the cognitive deficits. Further evidence for a role of *HUWE1* in ID comes from missense mutations in this gene that we detected in 3 ID families [2]. The patients of these families presented with a more severe form of ID with additional syndromic features. However, complete loss-of function of mouse *Huwe1* in the brain leads to neonatal lethality [3,4]. Deletion of *HUWE1* in the human population has never been reported.

The HECT, UBA and WWE domain containing 1 (*HUWE1*) gene encodes a ~500 kDa E3 ubiquitin ligase that targets its substrates for proteasomal degradation via poly-ubiquitination. Conditional *Huwe1* knockout studies in the mouse have proven the importance of this protein for neuronal development via its role in the regulation of Mycn, which is essential for the initiation of cell cycle exit and start of neuronal differentiation in the brain [3,4]. However, no studies have yet been performed on the effects of increased *HUWE1* expression *in vivo*. We used *Drosophila melanogaster* to investigate neuronal phenotypes and their underlying mechanisms/pathways. In the last decade, the fruitfly has emerged as a valuable model system for the study of ID [5,6]. In addition, it is especially suited for the investigation of increased expression in a tissue-specific manner because of the easy-to-use UAS-Gal4 system [7]. We generated *HUWE1* transgenic flies, which did not show severe neurological or behavioral alterations. However, at single axon resolution, increased *HUWE1* levels were found to disturb terminal branching of the dorsal cluster neurons (DCN), most likely by disturbing the canonical Wnt/ β-catenin pathway, a mechanism that has not been significantly investigated in association with ID.

## Materials and Methods

### Fly strains and generation of transgenic lines

Fly stocks were cultured on standard fly food and crosses were set up according to standard procedures. All experiments were performed in temperature-controlled incubators at 25°C (or 28°C when mentioned). To create the UAS-HUWE1 line, we cloned the human *HUWE1* cDNA (kindly obtained from V. Kalscheuer, Berlin) in the pUAST-attB vector followed by injection of this construct in *Drosophila* embryos by GenetiVision (www.genetivision.com). The construct was integrated in the fly genome via PhiC31-mediated site-specific integration at either insertion site VK37 at chromosome 2 or VK31 at chromosome 3 [8]. The transgenic flies will be referred to as UAS-HUWE1^VK37^ for insertion on 2L and UAS-HUWE1^VK31^ for insertion on 3L. Flies containing the AttP locus without a construct inserted were used as controls: control^VK31^ is y^1^w*;PBac{y^+^-attP-3B}VK00031, and control^VK37^ is y^1^w*;PBac{y^+^-attP-3B}VK00037. Other stocks used in this study are: nSyb-Gal4, ;elav-Gal4;elav-Gal4 Eyeless-Gal4 ;201Y-Gal4,UAS-DenMark,UAS-mCD8-GFP, 247-Gal4, UAS-dFz2GPI ;UAS-mCD8-GFP;atoGal4-14a,UAS-LacZ, Dsh<Dsh-GFP/CyO, Dsh<Dsh-GFP;UAS-HUWE1^VK31^, Dsh<Dsh-GFP/TM6, UAS-HUWE1^VK37^;Dsh<Dsh-GFP, UAS-arm^ACT^ and Canton S10. The KK101525 Dsh RNAi line was obtained from the Vienna *Drosophila* RNAi Center (VDRC; Vienna, Austria) [9]. Additional information on *Drosophila* stocks and genes can be found in Flybase (http://flybase.org/).

### Confirming the localization of UAS-HUWE1 constructs by PCR

The correct integration of the UAS-HUWE1 construct in the fly genome was confirmed by standard PCR with GoTaq polymerase (Promega) using primer HUWE1_ex24 localized in *HUWE1* in combination with either primer Dros_VK37 on chromosome 2L for UAS-HUWE1^VK37^, or primer Dros_VK31 on 3L for UAS-HUWE1^VK31^. Primer sequences are available in Table S1. 

### Expression analysis by RT-qPCR

Total RNA was isolated from fly heads via TRIzol (Invitrogen) – chloroform extraction, and cDNA was transcribed with the QuantiTect Reverse transcription Kit (Qiagen) on 1 µg RNA. qPCR was performed on the LightCycler 480 system (Roche), and each 15 µl reaction well contained 5 µl of a 1/60 dilution of cDNA, 0.5 µM of each primer and 1x SYBRgreen qPCR Master Mix (Roche). All samples were run in duplicate, and specificity of the PCR products was checked by a melt-curve analysis. The data were analyzed with the LC480 software followed by analysis in Microsoft Excel via the comparative ddCt method (Applied Biosystems, Foster City, CA), with normalization of the expression levels to the housekeeping gene *rp49*. In order to compare expression of the human *HUWE1* and the *Drosophila* homolog *CG8184*, we cloned the qPCR amplicons of *HUWE1*_ex73-74_for/rev and *CG8184*_for/rev together in the pGEM-T Easy vector (Promega). qPCR on this construct allowed us to compare the primer efficiency of both primer pairs and thus the accurate comparison of the expression levels of both genes. Primer sequences are available in Table S1.

### Immunohistochemistry on whole mount fly brain

Adult brains were dissected in 1x PBS and fixed with 4% formaldehyde in PBT (1x PBS + 0.3% Triton X-100) for 15 min. After two washes with PBT, the fixed brains were blocked for one hour in PAXDG buffer (PBT, 5% normal goat serum, 1% bovine serum albumin, 0.1% deoxycholate, 1% Triton X-100) and then incubated overnight at 4°C with primary antibodies diluted in 1x PAXDG. The following primary antibodies were used: mouse anti-GFP mAb 3E6 (Invitrogen cat. no. A11120) 1:1000, Rabbit polyclonal anti-DsRed (Clontech cat. no. 632496) 1:1000, mouse FasII 1D4 (DSHB) 1:50, and rabbit anti-GFP (Invitrogen cat. no. A11122) 1:500. This incubation was followed by 5 wash steps of 10 min each in PBT and a final incubation of 3 h at RT with the appropriate fluorescent secondary antibodies (Alexa 488, 555 or 647, Molecular probes; 1:500). After 6 washes of 10 min in PBT the samples were mounted in Vectashield Mounting Medium. Images of adult brains were acquired on a Leica TCS SP5 II confocal microscope system (Leica Microsystems) equipped with 458, 476, 488, 514, 543 and 633 nm lasers, and processed using ImageJ [10]. To quantify the branch number of DCN axons, we used the Simple Neurite Tracer plug-in in FiJi [11]. 

### Protein quantitation via Western Blotting

For determination of Dsh levels, we lysed four adult brains of each genotype in 10 µl 2x sample buffer (2% SDS, 10 mM Tris pH 6.8, 1 mM EDTA, 10% glycerol, 0.05% Bromophenolblue). The samples were boiled for 10 min at 99°C, after which 1 µl of DTT was added. The samples were run on a 4-12% NuPAGE Bis-Tris precast polyacrylamide gel (Invitrogen) in MOPS buffer and electrophoretically transferred to Hybond-C extra nitrocellulose membrane (GE Healthcare). After blocking for 1 h with 5% nonfat milk, the membrane was incubated overnight at 4°C with mouse anti-GFP (Roche) 1:1000 and mouse anti-actin JLA20 (DSHB) 1:100 as a loading control. After washing with PBT, the blot was probed with sheep anti-mouse IgG secondary antibody conjugated to HRP at 1:1000 dilution (GE Healthcare) for 1 h at RT. The bands were visualized with ECL western blotting detection reagents (GE healthcare) and digitally imaged with the Fujifilm LAS-300 Mini system (Life Science Systems). The experiment was repeated 4 times with flies kept at 25°C and 2 times with flies kept at 28°C. Quantitation was performed with ImageJ [10]. 

### Courtship conditioning assay

Double transgenic ;UAS-HUWE1^VK37^;UAS-HUWE1^VK31^ flies crossed to the mushroom body-specific 247-Gal4 driver were tested for learning and memory using the courtship conditioning assay as previously described [12]. Briefly, virgin males raised at 28°C were placed separately in food chambers together with a single mated female for a training period of 5 h. After training, all males were recovered and tested immediately to assess learning, or after 1 h to test short-term memory. All tests were carried out by pairing each male with a fresh mated female in a 1-cm courtship chamber for 10 min. The tests were videotaped and all assays were scored with customized tracking software from Actual Analytics (Edinburgh, UK). The mean Courtship Index (CI, the percentage of time spent on courtship during a 10 min interval) of trained males and of socially naïve males was used to calculate the Learning Index (LI), which is defined as the percent reduction in mean courtship activity in trained males compared with naïve males; LI = (CI_naive_–CI_trained_)/CI_naive_. No significant difference was found.

### Neuromuscular junction (NMJ) and electroretinogram (ERG) analysis

3^rd^ instar larvae grown at 28°C were dissected in PBS and fixed for 30 min in 3.7% paraformaldehyde. The fixed filets were incubated overnight at 4°C with mouse anti-brp (nc82, DSHB, 1:125 dilution). To visualize brp/nc82, secondary Alexa 488 goat-anti-mouse mAb was used (Invitrogen, 1:500 dilution). Subsequently, anti-discs large 1 (dlg1) (DSHB) pre-labeled with the Zenon Alexa Fluor 568 Mouse IgG1 labeling kit (Invitrogen) was applied. Acquired images were automatically processed and measured by an advanced in-house developed Fiji-based macro (Figure S1). The analysis was performed on NMJs of muscle 4 of at least 22 synaptic terminals.

ERG recordings were performed as described previously [13]. Briefly, flies were immobilized with Pritt glue on a glass slide after which a sharp glass reference electrode was inserted in the thorax, while a sharp recording electrode filled with 3 M NaCl was placed on the eye. Light flashes of 1 s were delivered using a halogen lamp. For each fly, 5 ERGs were recorded and we tested at least 5 flies per genotype. Data was digitized via pClamp and analyzed with Clampfit (Molecular Devices) and Excel (Microsoft).

### Statistical analysis

Data analysis was performed with Excel and GraphPad Prism 5. To compare the number of DCN axon branches in UAS-HUWE1, Dsh RNAi, Dsh^6^ and Dsh^1^ lines with control flies, we used 1-way ANOVA followed by Dunnett’s multiple comparison test. To compare branch number of UAS-HUWE1^VK31^, arm RNAi, UAS-arm^ACT^ and UAS-arm^ACT^;UAS-HUWE1^VK31^ lines with control flies and UAS-HUWE1^VK31^ with UAS-arm^ACT^;UAS-HUWE1^VK31^, we used 1-way ANOVA followed by Bonferroni’s multiple comparison test. Control^VK37^ and control^VK31^ flies were used as controls. For statistical analysis of dsh protein quantitation and NMJ experiments, we used the two-tailed unpaired Student’s t-test. In the courtship conditioning assay, non-parametric statistical comparison of *HUWE1* overexpressing and control flies was performed using a custom SAS script (SAS Institute, Inc.) to perform bootstrapping as described [14]. Briefly, CI values were randomly sampled with replacement to generate 10,000 hypothetical LIs, which were used to determine the 95% confidence interval of the difference between LI(control) and LI(knockdown).

## Results

### Generation of a fly model with HUWE1 overexpression

After cloning the cDNA of the human *HUWE1* gene in the pUAST-attB vector, site-specific integration via PhiC31 integrase was used to incorporate the construct in a well-defined AttP site on either the 2^nd^ or 3^rd^ chromosome of the fly. *HUWE1* expression can easily be regulated in a time- and tissue-specific manner by crossing the UAS-HUWE1 transgenic fly to a particular Gal4 driver line. The correct location of the UAS-HUWE1 construct in the fly genome was confirmed by PCR, and expression of human *HUWE1* was checked by RT-qPCR with three primer pairs distributed over the entire length of the mRNA. Both transgenic lines expressed human *HUWE1* from a single allele in the fly head at ~1.9 fold the expression level of the endogenous homolog *CG8184* when crossed to the pan-neuronal nSyb-Gal4 driver line. Both VK37 and VK31 lines were also crossed to generate a fly carrying the UAS-HUWE1 construct on both 2^nd^ and 3^rd^ chromosome. Crossing this double transgenic line to the neuron-specific ;elav-Gal4;elav-Gal4 driver at 28°C resulted in an expression level of ~1.4 times the expression of *CG8184*. In comparison, the same driver line combined with the UAS-HUWE1^VK31^ line resulted in a *HUWE1* expression that reached only about half the expression levels of the fly homolog *CG8184*. In ID patients with Xp11.22 duplications, the *HUWE1* mRNA levels in blood lymphocytes were increased 1.6- to 2.0-fold. Flies expressing the *HUWE1* via the neuron-specific elav-Gal4 or nSyb-Gal4 drivers from two alleles were viable and showed no overt morphological defects, which corresponds to the nonsyndromic phenotype of the patients. To study a potential effect of increased HUWE1 dosage on brain development and function in the fly, we first analyzed the flies for general neurological defects. 

### Learning and memory in the Courtship conditioning paradigm

As overexpression of *HUWE1* in humans was suggested to impair cognition to a level below an IQ of 70, we first subjected the transgenic flies to the courtship conditioning paradigm. The assay is based on the suppression of courtship behavior observed in male flies after they have been rejected by a pre-mated female [12]. Because the mushroom bodies (MB) are known to play an important role in this form of learning and memory and in cognition in general, we used the MB-specific 247-Gal4 line to drive *HUWE1* expression from the double transgenic ;UAS-HUWE1^VK37^;UAS-HUWE1^VK31^ line. The *HUWE1* line had been outcrossed for at least 7 generations into the CanS10 strain, which was used as a control in this assay.

**Figure 1:**
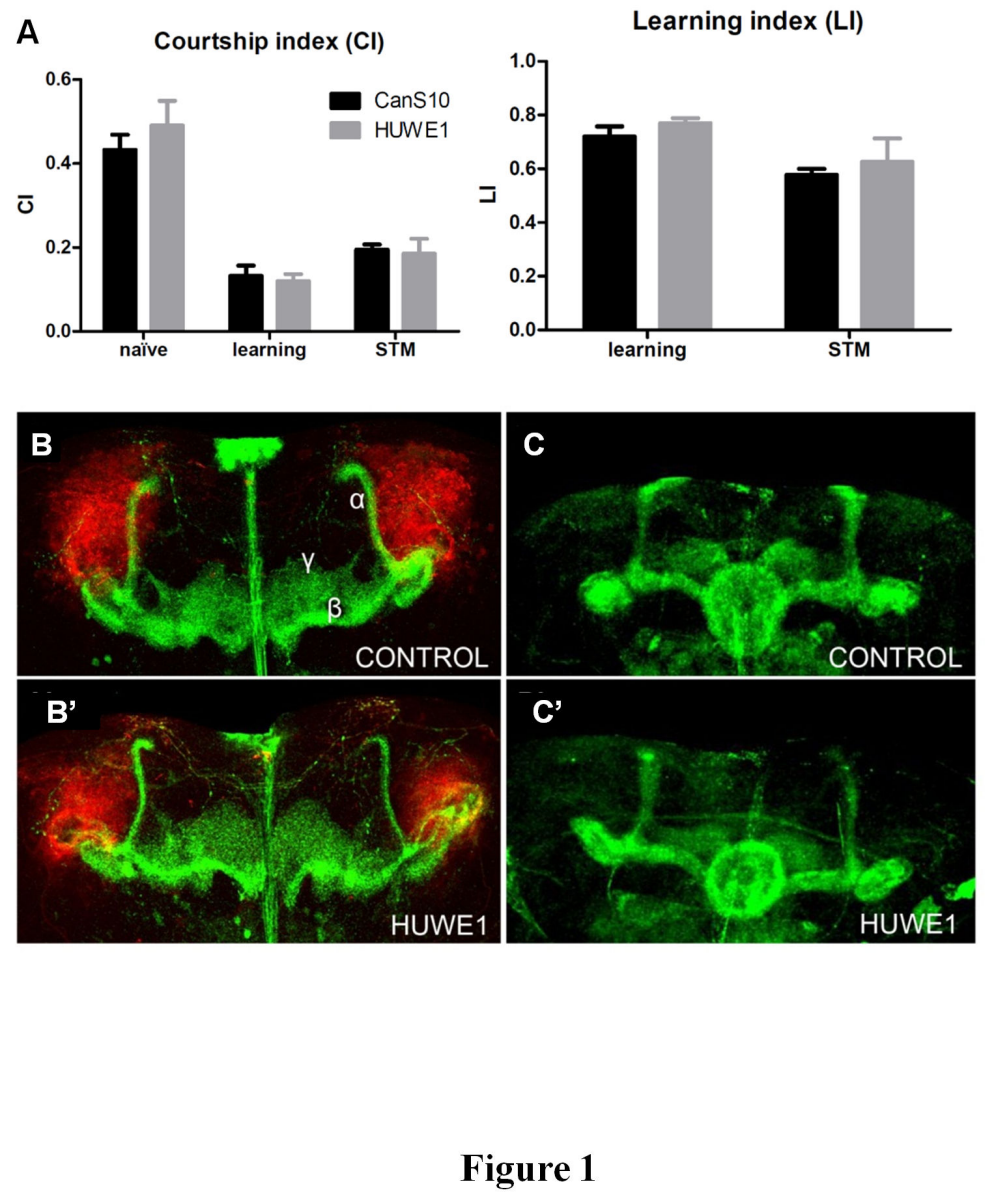
HUWE1 overexpression does not affect learning or MB development. (A) Short term memory (STM) was measured in the courtship conditioning paradigm in UAS-HUWE1^VK37^/+;UAS-HUWE1^VK31^/247-Gal4 males kept at 28°C. CI = courtship time / total time; LI = (CI_naïve_-CI_trained_) / CI_naïve_. Error bars represent the SEM. (B-C) Mushroom body analysis (B,B’) Control Canton S10 (CanS10) and UAS-HUWE1^VK31^ lines were crossed to the 201Y-Gal4,UAS-DenMark,UAS-mCD8-GFP driver line and kept at 25°C. The images are composites of a Z-projection of the confocal sections containing the axon lobes labeled by UAS-mCD8-GFP in green, and a Z-projection of the sections containing the calyx, labeled by DenMark in red. No gross abnormalities were observed in the axon lobes or the calyx. (C,C’) Expression of HUWE1 driven by the 247-Gal4 driver from the double transgenic ;UAS-HUWE1^VK37^;UAS-HUWE1^VK31^ line did not affect the morphology of the axon lobes when the flies were raised at 28°C. CanS10 flies were used as controls. The MB lobes were visualized by anti-FasII staining.

Socially naïve male flies were first trained by pairing them with a non-receptive mated female, and tested immediately following the training period to assess learning, or after 1 h to evaluate short term memory (STM). We then measured the mean Courtship Index (CI), which is the percentage of time spent on courtship during a 10 min interval, and calculated the Learning Index (LI). We found that learning in this courtship paradigm was not affected in flies with *HUWE1* overexpression in their MB. Also STM did not show any difference compared to the wild-type CanS10 flies (Figure 1A).

### Morphology of the mushroom body

To examine if overexpression of *HUWE1* had an effect on the structure of the nervous system we then investigated the MB for morphological alterations. The MB is formed by ~2500 Kenyon cells in each brain hemisphere that can be divided in 3 classes of neurons: γ neurons, α’/β’ neurons and α/β neurons. The γ neurons project a single horizontal axon, whereas the α’/β’ and α/β neurons axons branch in two projections, one horizontal and one vertical. Expression of *HUWE1* was driven by 201Y-Gal4, a line that expresses extensively in the γ neurons of the MB and also in a small subset of the α/β neurons [15]. These neurons were visualized by expression of UAS-driven membrane-associated MCD8-green fluorescent protein (GFP). No aberrations in the axon lobes were detected (Figure 1B-B’). In addition, we also specifically labeled the calyx, which is formed by the dendrites of the Kenyon cells, with the somatodendritic marker DenMark [16]. Also this dendritic structure did not present obvious morphological abnormalities (Figure 1B-B’). We confirmed the absence of morphological abnormalities in the axon lobes with the MB-specific 247-Gal4 line driving *HUWE1* expression from the double transgenic line ;UAS-HUWE1^VK37^;UAS-HUWE1^VK31^ (Figure 1C-C’). The lobes were visualized with anti-FasII staining but no significant differences compared to the controls were detected. Since the MB is a dense structure formed by a large number of neurons, it is possible that more subtle aberrations remained unnoticed.

### Analysis of the neuromuscular junctions and basal neurotransmission

Next, we examined the larval neuromuscular junction (NMJ), which is the most accessible synapse in the fly [17]. The *Drosophila* NMJ shares important features with central excitatory synapses in the vertebrate brain, as the NMJ is a glutamatergic synapse with ionotropic glutamate receptors that are homologous to those of humans [18]. We used the pan-neuronal ;UAS-dicer2; elav-Gal4 line to drive *HUWE1* expression from the double transgenic ;UAS-HUWE1^VK37^;UAS-HUWE1^VK31^ line and examined the NMJ of muscle 4 in wandering 3^rd^ instar larvae. No morphological abnormalities were detected in the NMJ: the NMJ area, perimeter and length were not affected and there were no differences in the number of branches and branching points (Table S2). We also investigated the number of active zones, which are the presynaptic sites of neurotransmitter release. The number of active zones, visualized by anti-nc82 staining, was not affected by increased *HUWE1* levels (Table S2).

Basal neurotransmission can be detected by recording an electroretinogram (ERG), which assesses if synaptic transmission between photoreceptors and their post synaptic targets in the lamina in response to a light flash occurs and is synchronized. An ERG shows the voltage difference between the retina and the rest of the body during a short (1 s) light pulse. Adult flies expressing *HUWE1* in their entire nervous system by the nSyb-Gal4 driver did not present any abnormalities in the ERG profile, indicating that neurotransmission and neuronal connectivity are largely unaffected (Figure S2). However, defects in synaptic plasticity cannot be excluded.

### Axon branching in the dorsal cluster neurons

As our analyses above did not reveal any major alterations upon moderate *HUWE1* overexpression, we decided to investigate if defects at a more sensitive level might explain why increase in *HUWE1* levels causes cognitive problems in ID patients. For this we made use of the dorsal cluster neurons (DCNs) as a model because subtle changes can be detected at single axon resolution. The DCN consist of a small cluster of neurons whose axons grow in a very stereotypical pattern allowing the detection of subtle defects in axon outgrowth and branching. *HUWE1* was expressed specifically in these neurons by means of the *atoGal4-14a* driver line. This driver starts to be expressed in early third instar larvae, which is shortly before the DCNs begin to extend their axons towards the optic lobes, and continues to be active during metamorphosis and into adult life [19,20]. The neurons were visualized by expression of the membrane-associated mCD8-GFP. 

**Figure 2:**
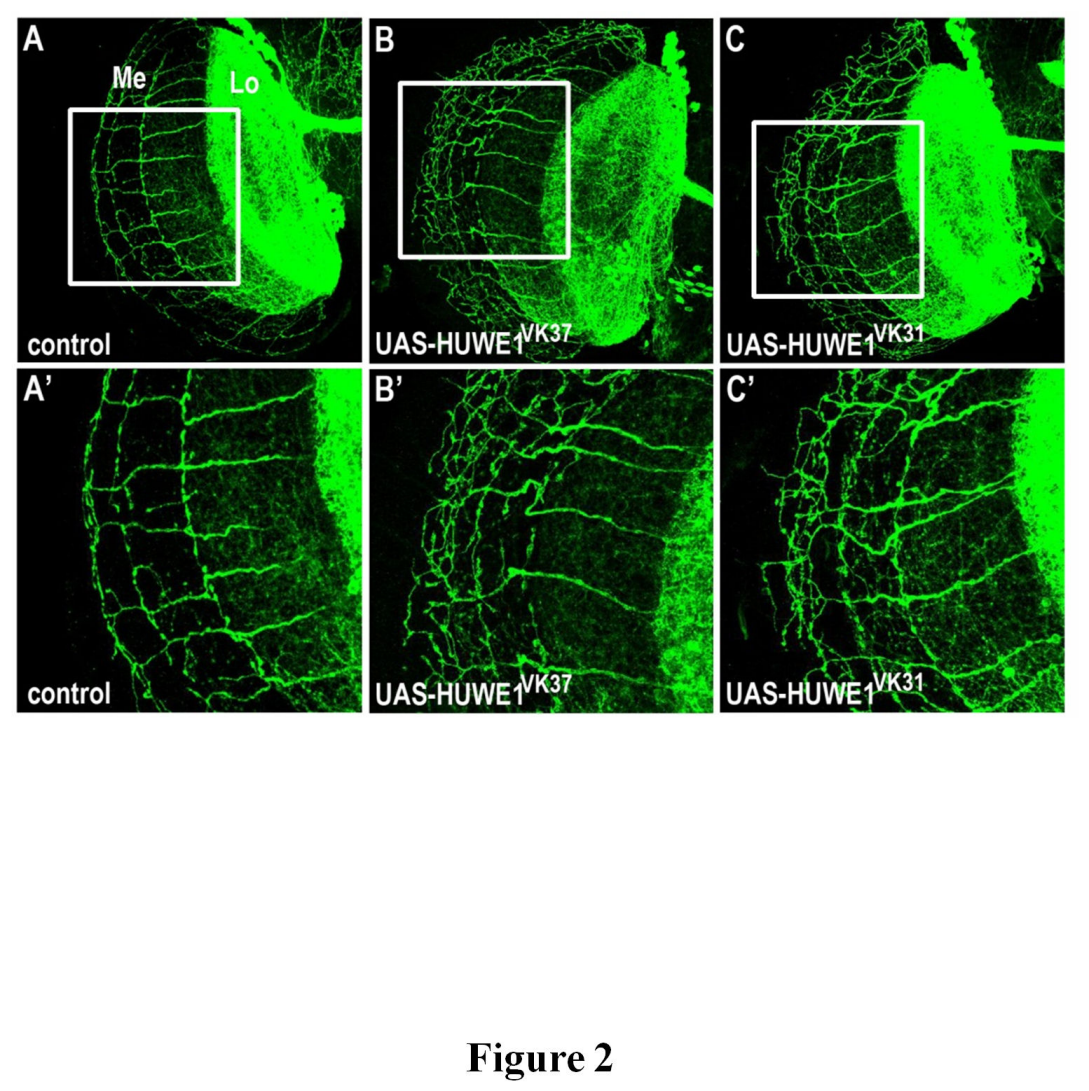
HUWE1 affects DCN axon branching. (A-C’) Axon projections of the DCN in the optic lobe, visualized via staining against mCD8-GFP. Lo = lobula, Me = Medulla. (A) Representative image of a control brain: w;UAS-mCD8-GFP/+;control^VK31^/atoGal4-14a,UAS-LacZ. (A’) Magnification of the branching area in the white square shown in panel A. (B,C) Overexpression of HUWE1 in w;UAS-HUWE1^VK37^/UAS-mCD8-GFP;atoGal4-14a,UAS-LacZ/+ and w;UAs-mCD8-GFP/+;UAS-HUWE1^VK31^/atoGal4-14a,UAS-LacZ flies does not affect axon number in the medulla, but leads to increased axon branching at the 3^rd^ branching point. (B’,C’) Magnification of the branching area in the white squares shown in panels B and C.

In wild-type adult flies, 11-12 parallel axons cross the optic chiasm between lobula and medulla, after which they branch to form a stereotypical grid-like structure [20]. The number of axons crossing the optic chiasm is not affected in flies with increased *HUWE1* levels, with an average of 11.3 axons innervating the medulla for UAS-HUWE1^VK37^ versus 11.1 in control^VK37^, and 11.7 axons in UAS-HUWE1^VK31^ versus 11.9 in control^VK31^. However, the branching pattern in the medulla was disturbed giving rise to an increased number of branches at the 3^rd^ branching point of the grid-like structure (Figure 2). The effect was seen with UAS-HUWE1^VK37^ as well as with UAS-HUWE1^VK31^, indicating that the phenotype is specifically due to increased levels of *HUWE1* and does not occur as a result of position effects caused by the insertion of the construct in the fly genome. Moreover, overexpression of another seemingly dosage-sensitive ID gene *GDI1* that we tested with the same DCN-specific driver did not affect axon branching (data not shown), pointing to specificity of the phenotype for increased *HUWE1* levels. 

**Figure 3:**
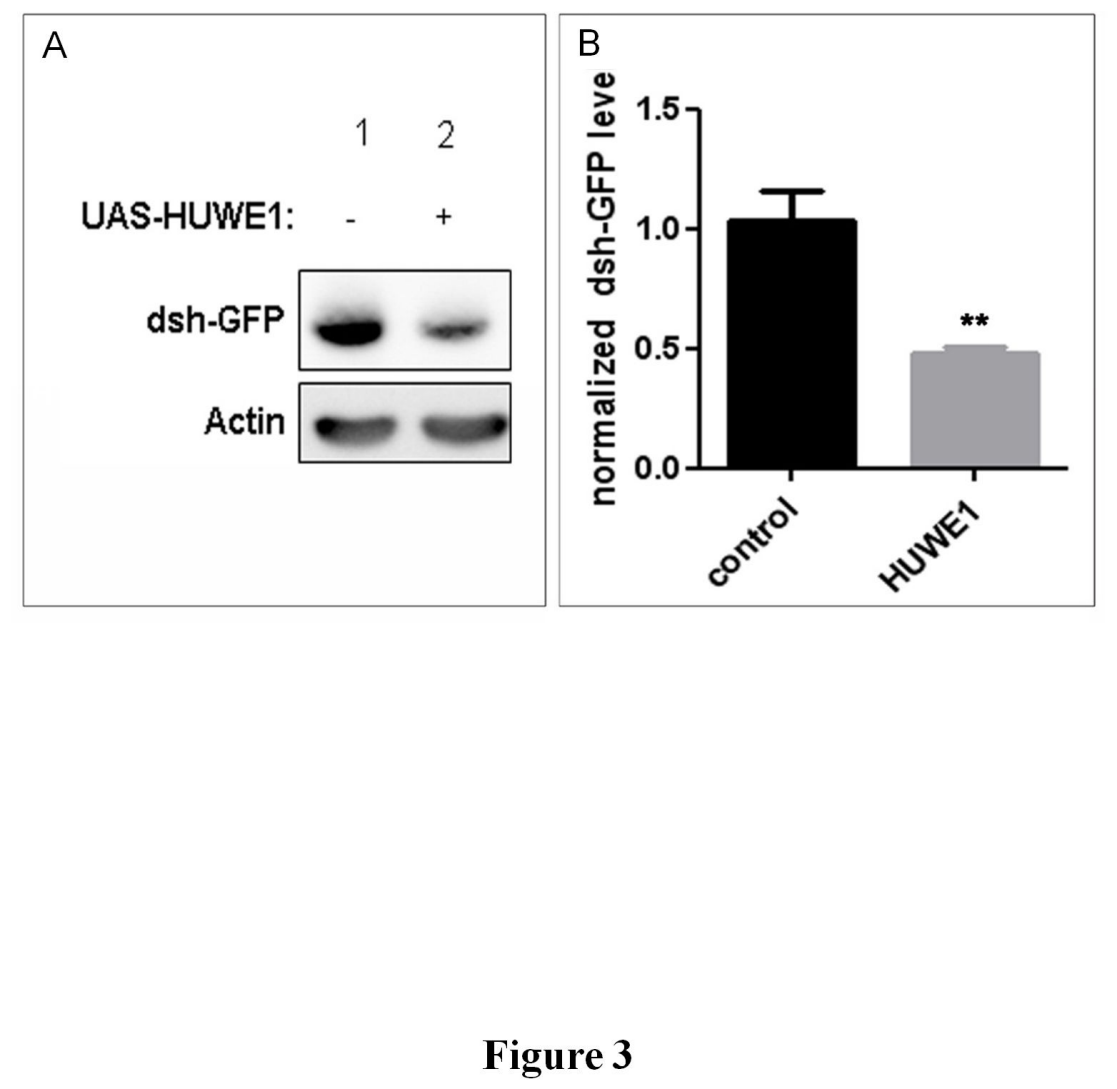
HUWE1 overexpression reduces dsh levels. (A) HUWE1 expression was driven by the pan-neuronal nSyb-Gal4 driver, and whole-brain lysates were subjected to SDS-PAGE. Dsh-GFP was visualized with an anti-GFP antibody, Actin was used as loading control. Lane 1: dsh<dsh-GFP/+;nSyb-Gal4/+; Lane 2: dsh<dsh-GFP/+;UAS-HUWE1^VK31^/nSyb-Gal4. (B) Quantitation of dsh-GFP levels, normalized to actin. The graph represents the average of 4 biological repeats with SEM. Dsh-GFP levels are significantly reduced in flies with HUWE1 overexpression (** p<0.01, Student’s t-test). Control = dsh<dsh-GFP/+;nSyb-Gal4/+; HUWE1 = dsh<dsh-GFP/+;UAS-HUWE1^VK31^/nSyb-Gal4.

**Figure 4:**
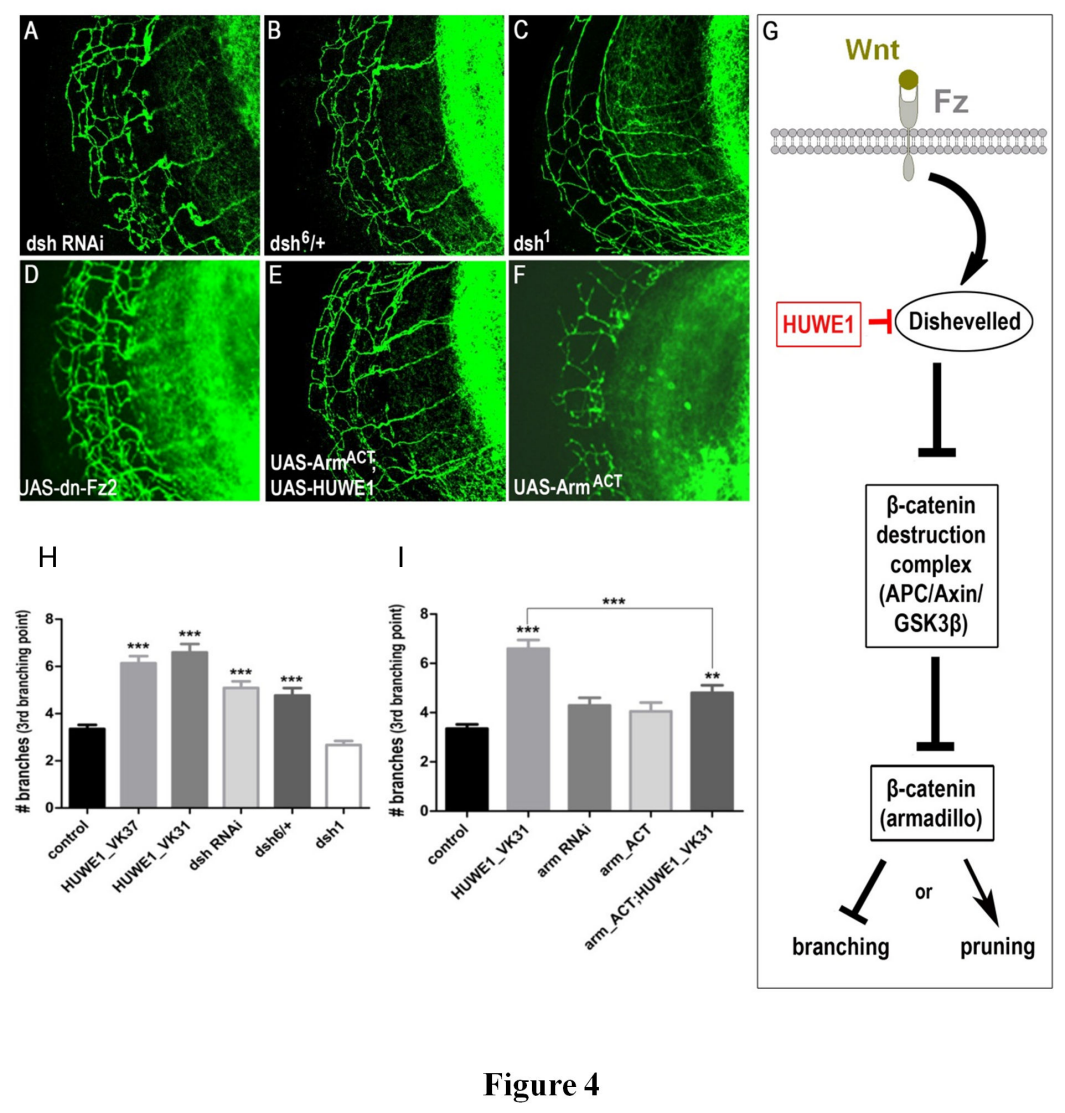
The Wnt/β-catenin pathway is involved in the disturbed DCN branching. (A,B) Reduced dsh levels in w;UAS-dsh-RNAi/UAS-mCD8-GFP;atoGal4-14a,UAS-LacZ/+ and heterozygous null mutant dsh^6^/+;UAS-mCD8-GFP/+;atoGal4-14a,UAS-LacZ/+ animals also led to an increased axon branching at the 3^rd^ branching point. (C) DCN axon branching is normal in dsh^1^;UAS-mCD8-GFP/+;atoGal4-14a,UAS-LacZ/+ males, which are only mutant in the DEP domain responsible for activation of the non-canonical pathway. (D) DCN axon branching is equally affected in dominant negative Fz2;UAS-dn-Fz2/UAS-mCD8-GFP;atoGal4-14a,UAS-LacZ/+ flies. (E) Combined expression of HUWE1 and *Arm* ^*ACT*^ in w;UAS-Arm^ACT^/UAS-mCD8-GFP;UAS-HUWE1^VK31^/atoGal4-14a,UAS-LacZ flies partially rescues the branching phenotype, although the number of branches is not completely reverted to wild-type levels. (F) Expression of a constitutively active *Arm* mutant in w;UAS-Arm^ACT^/UAS-mCD8-GFP;atoGal4-14a,UAS-LacZ/+ animals results in a reduced branching phenotype. (G) Model showing the association of HUWE1 with the Wnt/β-catenin pathway and its effect on axon branching and/or pruning, as evidenced by our data. (H,I) Quantitation of the axon branching levels at the 3^rd^ branching point of the DCNs in the medulla. We evaluated 20-25 neurons from at least 5 different brains per genotype. Error bars represent standard error of the mean (SEM) (*** p<0.001, ** p<0.01).

Next, we investigated the pathway that could affect axon branching upon *HUWE1* overexpression. Huwe1 has recently been demonstrated to ubiquitinate dishevelled (dsh in flies, Dvl in mammals), a major component of the Wnt/β-catenin pathway (V. Bryja, personal communication). To test whether increased *HUWE1* expression in the fly brain, driven by the pan-neuronal nSyb-Gal4 driver, had an effect on dsh levels, we expressed GFP-tagged dsh under its own promoter, allowing us to visualize dsh-GFP with an anti-GFP antibody. Western blot detected a 50% reduction in Dsh-GFP levels in dshdsh-GFP/+;UAS-HUWE1^VK31^/nSyb-Gal4 flies compared to the controls (Figure 3). These data thus suggest that the Wnt/β-catenin pathway is also involved in the fly model presented here. If so, lower levels of the *Drosophila* dsh should result in a similar increased branching phenotype. Indeed, flies targeting dsh expression via RNA interference (RNAi) presented a significant increase in branch number at the 3^rd^ branching point of the DCNs, strikingly similar to *HUWE1* overexpressing conditions (Figure 4A). The same phenotype was observed in flies heterozygous for *dsh* ^6^, a null allele of *dsh* (Figure 4B). Homozygous null mutants could not be tested due to embryonic lethality. We also investigated the effect of the *dsh* ^1^ mutant on DCN axon branching. This mutant is deficient only in the activity of its DEP domain (Dishevelled, Egl-10, Pleckstrin), which is important for the activation of the non-canonical JNK signaling pathway by regulation of Rho family GTPase proteins. In contrast, it leaves the DIX domain (Dishevelled and Axin) intact, which is specifically required for the activation of the canonical β-catenin pathway [21,22]. DCN axon branching at the 3^rd^ branching point was not affected in *dsh* ^1^ mutant males (Figure 4C), indicating that activation of the non-canonical pathway via the DEP domain does not play a role in this branching process. A second line of evidence for the involvement of the Wnt/β-catenin pathway was observed upon expression of a dominant-negative mutant form of the Wnt-receptor *frizzled2* (*fz2*), which resulted in a similar disturbed DCN branching phenotype as observed for *HUWE1* overexpression (Figure 4D). As illustrated in Figure 4G activation of the *frizzled* (*fz*) receptor upon binding of its Wnt ligand will lead to inhibition of the β-catenin destruction complex via activation of dsh. β-catenin can then activate its target genes, which could in this case either stop the branching process or initiate pruning of excessive branches. Expression of the dominant-negative *fz2* prevents this activation and thus causes the observed increase in axon branch number. Finally, the involvement of the Wnt/β-catenin pathway was investigated via the *Drosophila* homolog of β-catenin, armadillo (arm). We were able to partially rescue the increased branching phenotype by combining *HUWE1* overexpression with expression of a constitutively active mutant of *arm* (Figure 4E). This mutant can no longer be inactivated by the β-catenin destruction complex, and as a consequence it is not affected by the HUWE1-driven breakdown of dsh. These data are in agreement with the expression of a constitutively active *arm*, which on its own resulted in a reduced branching phenotype (Figure 4F). Quantification of branching relative to the controls is shown in Figures 4H-I, which also shows that RNAi for *arm* does not affect branching. In conclusion, we provide evidence that overexpression of *HUWE1* lead to increased breakdown of dsh, causing the β-catenin destruction complex to remain more active and thus reduce the activation of β-catenin-dependent genes (Figure 4G).

## Discussion

In this study we developed a *Drosophila melanogaster* model to investigate the effect of increased *HUWE1* expression on the developing nervous system. Quantitation of *HUWE1* levels indicated that expression in the fly brain was increased in a range similar to what is seen in the patients, who presented 1.6- to 2-fold increase levels compared to controls [2]. For instance, the pan-neuronal ;elav-Gal4;elav-Gal4 driver line resulted in a *HUWE1* expression that reached about half the levels of the fly homolog *CG8184*. Assuming that human HUWE1 and *Drosophila* CG8184 perform the same functions, the transgenic fly has a combined *HUWE1-CG8184* level that is 1.5 fold higher compared to that of the endogenous *CG8184* expressed in control flies. This increased dosage is in the same range as the 1.5- to 1.8-fold increase described for dap160, synj and nla in a fruitfly model for Down syndrome. Even this moderate overexpression of Down syndrome candidate proteins led to defects in synaptic development and activity at the NMJ [23]. These ratios measured in the flies probably underestimate the *HUWE1* expression levels in neurons, as these are determined using RNA extracts from whole heads. 

We previously provided strong evidence that a 1.6- to 2.0-fold increase of *HUWE1* expression in human results in a mild cognitive deficit. Hence, we first analyzed whether flies overexpressing *HUWE1* showed impaired learning as well. However, we did not observe defects in learning and memory in the courtship conditioning paradigm when *HUWE1* levels were specifically increased in the MB, a large neuropil in the central brain known to be involved in learning and memory. *HUWE1* levels might need to be increased in the entire courtship circuit to cause a phenotype. Furthermore, no broad structural abnormalities were noticed in the MB. This finding could be in accordance with the fact that MRI brain imaging and CT scans of patients with *HUWE1* duplications did not reveal gross structural aberrations [2]. Morphology of the NMJs and neurotransmission, measured as ERGs, were unaffected as well in flies with a pan-neuronal increased expression of *HUWE1*. These data are in agreement with the lack of additional clinical features in the patients as *HUWE1* is a ubiquitously expressed gene. 

By looking for more subtle alterations however, we detected a significant increase in branch numbers at the axon terminals of the dorsal cluster neurons. The DCNs are a group of ~40 neurons that are part of the *Drosophila* visual system. Their cell bodies are located in the dorso-lateral region of the central brain, from where they project their axons to the controlateral side of the brain to innervate the optic lobe [15,16]. Our data suggest that this altered branching phenotype was caused via a negative regulatory effect of HUWE1 on dsh, a key component of the Wnt signaling pathway that transmits Wnt signals from the *fz* receptors to downstream effectors. Though Wnt signaling plays an important role in a wide range of biological processes [24], it has been associated with terminal branching of neurons too. Wnt5a and Wnt3 have previously been implicated to regulate the axon branching in the mouse sympathetic and spinal sensory dorsal root ganglia neurons, respectively [25,26]. In *Drosophila*, axon branching of the MB neurons was shown to be regulated by Wnt signaling [27]. Altered Wnt signaling has also been linked to synaptic plasticity in the mouse [28] and to other neurological disorders such as Alzheimer’s disease [29], Williams syndrome [30] and schizophrenia [31,32], as well as neurodevelopmental abnormalities including neural tube defects and agenesis of the corpus callosum [24]. Interestingly, ubiquitination has been shown to play an important role in the regulation of Wnt signaling by affecting many different steps in the pathway, as reviewed by Tauriello and Maurice [33]. In fact, the E3 ubiquitin ligase Nedd4 was demonstrated to promote axon branching in Xenopus retinal ganglion cells [34]. Recently, Huwe1 was identified as a conserved negative regulator of the Wnt/β-catenin pathway acting at the level of Dvl (V. Bryja, personal communication) and conditional knock down of different key components of the Wnt pathway, including arm, in the MB of adult flies disrupted long term memory in the olfactory behavioral paradigm [35]. Finally, HUWE1 was identified as an interactor of the ubiquitin E3 ligase RNF146, which was demonstrated to promote Wnt signaling [36].

We could ameliorate the increased branching phenotype caused by *HUWE1* overexpression upon co-expressing a constitutively active form of the β-catenin homolog *arm*, indicating that the canonical Wnt/β-catenin pathway is affected by increased *HUWE1* levels. However, as the phenotype was not completely rescued, other Wnt signaling pathways might play a role as well. It is of significant interest to note that two nonsense and one frameshift mutation in the *CTNNB1* gene that encodes the β-catenin protein have recently been described in patients with very similar clinical features including severe ID with absent or very limited speech, microcephaly and spasticity [37]. Regarding signaling, we can exclude involvement of JNK signaling, as the *dsh* ^1^ mutant, which is specifically mutated in the DEP domain necessary for activation of JNK signaling [21], did not affect terminal axon branching. The Wnt-dsh-JNK signaling pathway has been shown to regulate axon extension and retraction in the DCNs, and both *dsh* ^6^ and *dsh* ^1^ mutants affect this pathway causing a reduction in the number of DCN axons reaching the medulla [20]. Although western blot indicated that HUWE1 reduces the dsh protein levels, we did not see an effect on the DCN axon numbers in the medulla. The effect of HUWE1 on terminal branching without affecting axon outgrowth via JNK signaling could be explained by the existence of different dsh pools, with HUWE1 possibly selectively affecting one pool without targeting the other. Dvl/dsh has been shown to shuttle between the cytoplasm and the nucleus [38-42], and interestingly, disruption of the nuclear localization signal of dsh specifically impairs the canonical β-catenin pathway [41]. Another study indicated that this nuclear Dvl is crucial for the formation of a stable complex between β-catenin and T cell factor (TCF), via which it can affect β-catenin-dependent transcription [39]. If overexpression of *HUWE1* specifically affects the nuclear dsh pool, this could account for the absence of non-canonical pathway-dependent phenotypes. Alternatively, the axon branching could be more sensitive to changes in dsh levels compared to the axon extension. The fact that dsh RNAi flies still have on average 11.3 axons projecting to the medulla is in agreement with this hypothesis.

To conclude, increased expression of *HUWE1* did not cause major structural defects in the brain of *Drosophila*, but the terminal axon branching of the DCNs was severely disturbed. Our results suggest that this branching phenotype is caused by enhanced breakdown of dsh through increased levels of HUWE1 resulting in reduced activation of the canonical Wnt/β-catenin pathway. It therefore is tempting to speculate that a similar process is acting in ID patients with duplication of *HUWE1*, linking Wnt/β-catenin signaling to memory formation. 

## Supporting Information

Figure S1 **Automated analysis of the NMJ.** Visualization of the NMJ analysis as generated by an in-house developed ImageJ/FiJi-based macro. Anti-Dlg1 staining is shown in red. The blue line traces the length and branches of the NMJ, whereas the yellow line on the outside of the NMJ visualizes the NMJ perimeter. The white dots represent the active zones (as determined by anti-nc82 staining).
(DOCX)

Figure S2 **Neurotransmission is unaffected in flies with pan-neuronal *HUWE1* overexpression.** ERGs from control^VK31^/nSyb-Gal4, UAS-HUWE1^VK31^/nSyb-Gal4, control^VK37^/+;nSyb-Gal4/+ and HUWE1^VK37^/+;nSyb-Gal4/+ flies. The arrowheads in control^VK31^ indicate the on and off transients.
(DOCX)

Table S1 **Primers used for PCR and RT-qPCR.**
(DOCX)

Table S2 **Quantification of NMJ parameters in 25 controls and 30 HUWE1 transgenes.** NMJ parameters were automatically quantified by an in-house developed ImageJ/FiJi-based macro, as visualized in Figure S1. NMJ area was normalized to muscle area. No parameters reached a p-value < 0,01 (Student’s t-test).
(DOCX)
