# Correction: Ubiquitin Ligase HUWE1 Regulates Axon Branching through the Wnt/β-Catenin Pathway in a *Drosophila* Model for Intellectual Disability

**DOI:** 10.1371/annotation/5a4ac42e-a148-4a89-b148-e47de0d72d16

**Published:** 2014-01-03

**Authors:** Joke Vandewalle, Marion Langen, Marlen Zschaetzsch, Bonnie Nijhof, Jamie M. Kramer, Hilde Brems, Marijke Bauters, Elsa Lauwers, Mohammed Srahna, Peter Marynen, Patrik Verstreken, Annette Schenck, Bassem A. Hassan, Guy Froyen

The name of the third author is incorrect. The correct name is: Marlen Zschätzsch. 

The correct Citation is: Vandewalle J, Langen M, Zschätzsch M, Nijhof B, Kramer JM, et al. (2013) Ubiquitin Ligase HUWE1 Regulates Axon Branching through the Wnt/β-Catenin Pathway in a *Drosophila* Model for Intellectual Disability. PLoS ONE 8(11): e81791. doi:10.1371/journal.pone.0081791

