# Supplementary figures and images for: Ubiquitin Ligase HUWE1 Regulates Axon Branching through the Wnt/β-Catenin Pathway in a Drosophila Model for Intellectual Disability
Source: PLoS One. 2013 Nov 26;8(11):e81791. doi: 10.1371/journal.pone.0081791 (PMC3841167; doi:10.1371/journal.pone.0081791)

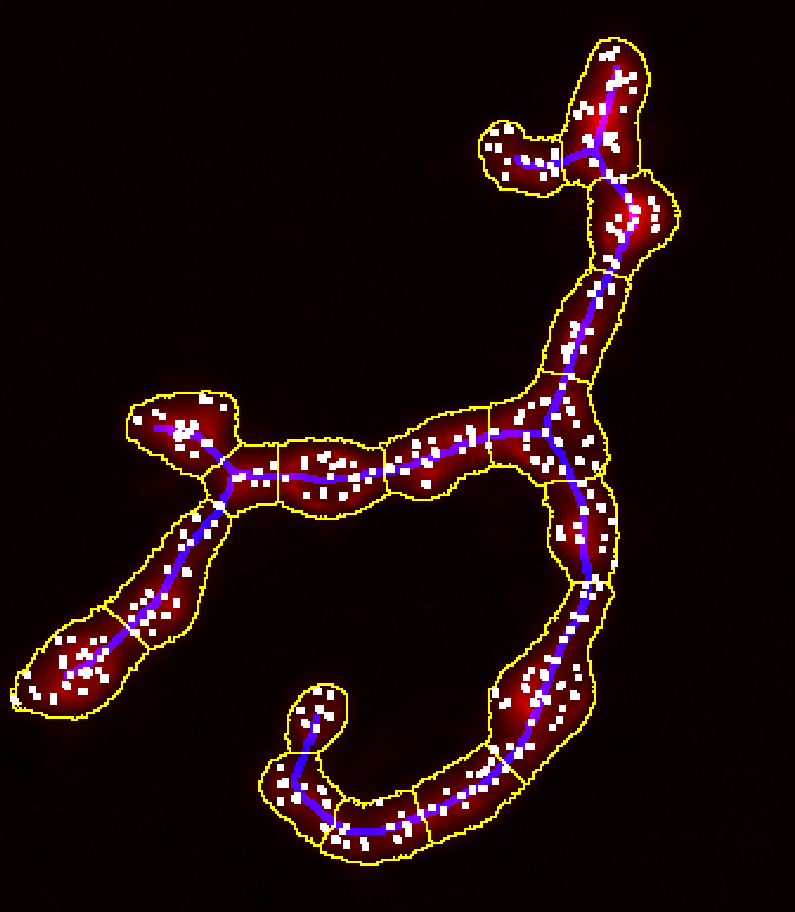


**Figure S1**

Supplement: Figure S1 — Automated analysis of the NMJ. Visualization of the NMJ analysis as generated by an in-house developed ImageJ/FiJi-based macro. Anti-Dlg1 staining is shown in red. The blue line traces the length and branches of the NMJ, whereas the yellow line on the outside of the NMJ visualizes the NMJ perimeter. The white dots represent the active zones (as determined by anti-nc82 staining). (DOCX) [file pone.0081791.s001.docx]

**
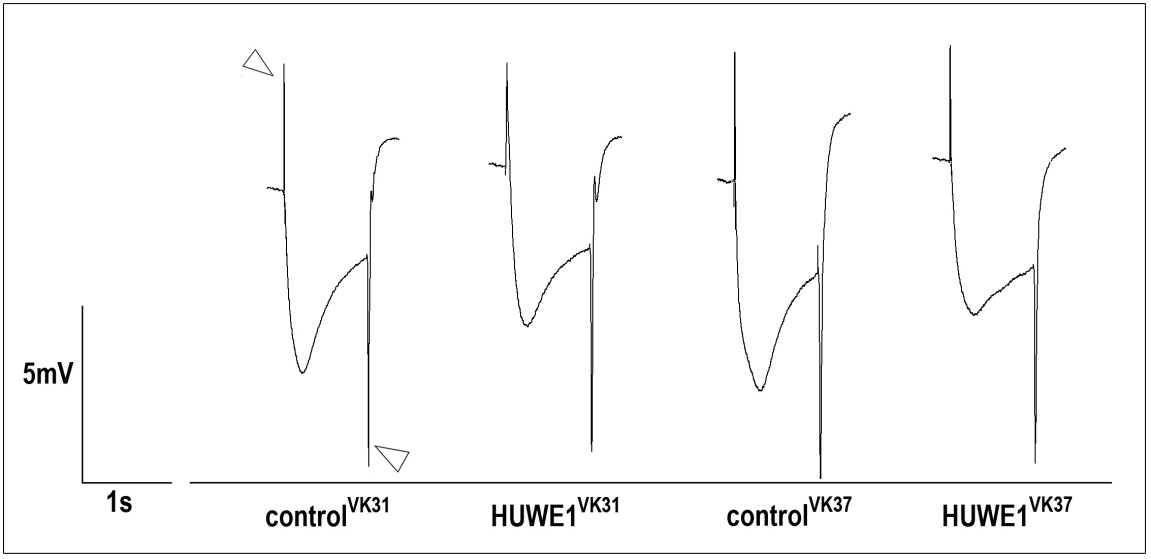
**

Figure S2

Supplement: Figure S2 — Neurotransmission is unaffected in flies with pan-neuronal HUWE1 overexpression. ERGs from controlVK31/nSyb-Gal4, UAS-HUWE1VK31/nSyb-Gal4, controlVK37/+;nSyb-Gal4/+ and HUWE1VK37/+;nSyb-Gal4/+ flies. The arrowheads in controlVK31 indicate the on and off transients. (DOCX) [file pone.0081791.s002.docx]
