# Supplementary material for: Ubiquitin Ligase HUWE1 Regulates Axon Branching through the Wnt/β-Catenin Pathway in a Drosophila Model for Intellectual Disability
Source: PLoS One. 2013 Nov 26;8(11):e81791. doi: 10.1371/journal.pone.0081791 (PMC3841167; doi:10.1371/journal.pone.0081791)
Supplement: Table S1 — Primers used for PCR and RT-qPCR. (DOCX) [file pone.0081791.s003.docx]

Table S1. Primers used for PCR and RT-qPCR

| Primer name | Primer sequence |
| --- | --- |
| HUWE1_ex24 | 5'-CTATCCAGGAGTGCTCTGAA |
| Dros_VK37 | 5'-TCGACTTTGCTCAACACACA |
| Dros_VK31 | 5'-CGGGGTTTCGGTTACTCTTT |
| rp49_for | 5'-TATGCTAAGCTGTCGCACAAAT |
| rp49_rev | 5'-CTGTCCCTTGAAGCGGC |
| CG8184_for | 5'-TGGAAGAACCGCTTCTACAT |
| CG8184_rev | 5'-CATACCACTCGCGCAACA |
| HUWE1_ex7-8_for | 5'-GGGATCTGACAAGAGGAC |
| HUWE1_ex7-8_rev | 5'-CCAAAGCCATTCTCCTTTCCA |
| HUWE1_ex23-24_for | 5'-ACAGGCCATGCAGAGCTTTAA |
| HUWE1_ex23-24_rev | 5'-ATACGTTCCTCTGTACCAACAACCT |
| HUWE1_ex73-74_for | 5'-CTGAGGCTGATGCCATTATACA |
| HUWE1_ex73-74_rev | 5'-CTGGCTAGACTCCGACG |
