# Supplementary material for: Ubiquitin Ligase HUWE1 Regulates Axon Branching through the Wnt/β-Catenin Pathway in a Drosophila Model for Intellectual Disability
Source: PLoS One. 2013 Nov 26;8(11):e81791. doi: 10.1371/journal.pone.0081791 (PMC3841167; doi:10.1371/journal.pone.0081791)
Supplement: Table S2 — Quantification of NMJ parameters in 25 controls and 30 HUWE1 transgenes. NMJ parameters were automatically quantified by an in-house developed ImageJ/FiJi-based macro, as visualized in Figure S1. NMJ area was normalized to muscle area. No parameters reached a p-value < 0,01 (Student’s t-test). (DOCX) [file pone.0081791.s004.docx]

**Table S2. Quantification of NMJ parameters in 25 controls and 30 HUWE1 transgenes**

NMJ parameters were automatically quantified by an in-house developed ImageJ/FiJi-based macro, as visualized in Fig. S1. NMJ area was normalized to muscle area. No parameters reached a p-value < 0,01 (Student’s t-test).

|  | **Canton S10** | **HUWE1** | **P-value** |
| --- | --- | --- | --- |
| **Muscle area** | 60,122 µm² | 54,381 µm² | 0.016 |
| **NMJ area** | 382 µm² | 365 µm² | 0.495 |
| **normalized NMJ area** | 0.64 | 0.67 | 0.737 |
| **NMJ perimeter** | 2,020 µm | 2,067 µm | 0.697 |
| **NMJ length** | 121 µm | 131 µm | 0.113 |
| **# branches** | 6.79 | 6.17 | 0.519 |
| **# branching points** | 2.33 | 1.79 | 0.200 |
| **# active zones** | 247 | 248 | 0.841 |
